# Comparison of mitochondrial gene expression and polysome loading in different tobacco tissues

**DOI:** 10.1186/s13007-017-0257-4

**Published:** 2017-12-13

**Authors:** Muhammad Waqar Hameed, Ilona Juszczak, Ralph Bock, Joost Thomas van Dongen

**Affiliations:** 1 Max Planck Institute of Molecular Plant Physiology, Am Mühlenberg 1, 14476 Potsdam-Golm, Germany; 2 Dr. Panjwani Center for Molecular Medicine and Drug Research, International Center for Chemical and Biological Sciences, University of Karachi, Karachi, 75270 Pakistan; 3 Molecular Physiology, University of Bonn, Kirschallee 1, 53115 Bonn, Germany; 4 Institute of Biology I, RWTH Aachen University, Worringerweg 1, 52056 Aachen, Germany

**Keywords:** Plant mitochondria, Transcription, Translation, Mitochondrial microarray, Mitochondrial ribosomes

## Abstract

**Background:**

To investigate translational regulation of gene expression in plant mitochondria, a mitochondrial polysome isolation protocol was established for tobacco to investigate polysomal mRNA loading as a proxy for translational activity. Furthermore, we developed an oligonucleotide based microarray platform to determine the level of *Nicotiana tabacum* and *Arabidopsis thaliana* mitochondrial mRNA.

**Results:**

Microarray analysis of free and polysomal mRNAs was used to characterize differences in the levels of free transcripts and ribosome-bound mRNAs in various organs of tobacco plants. We have observed higher mitochondrial transcript levels in young leaves, flowers and floral buds as compared to fully expanded leaves and roots. A similar pattern of abundance was observed for ribosome-bound mitochondrial mRNAs in these tissues. However, the accumulation of the mitochondrial protein COX2 was found to be inversely related to that of its ribosome-bound mRNA.

**Conclusions:**

Our results indicate that the association of mitochondrial mRNAs to ribosomes is largely determined by the total transcript level of a gene. However, at least for *Cox2,* we demonstrated that the level of ribosome-bound mRNA is not reflected by the amount of COX2 protein.

**Electronic supplementary material:**

The online version of this article (10.1186/s13007-017-0257-4) contains supplementary material, which is available to authorized users.

## Background

Mitochondria evolved when a free-living α-proteobacterium was engulfed by a single-celled protist [1, 2]. The engulfed cell was not digested but rather domesticated by the host cell to establish an endosymbiotic association. During the course of evolution, the mitochondrial genome was significantly reduced, due to the loss of genes or their transfer to the nuclear genome of the host cell. Consequently, protein products from genes that are now expressed in the nucleus are imported into the mitochondria [3–5]. Regardless of the many gene translocations, mitochondria in all multicellular organisms and most lineages of protists retained their own genome [6–8]. Compared to the organization and structure of mitochondrial genomes in animals and fungi, the plant mitochondrial genome is very complex [7, 9]: it can vary between 180 kb and 11.3 Mb in size, has numerous direct and inverted repeats; contains a number of (conserved) open reading frames of unknown function, as well as several gene sequences acquired from plastids or the nuclear genome [10–12]. The genes that are retained in the plant mitochondrial genome encode three ribosomal RNAs, 15–20 transfer-RNAs and approximately 30 proteins that include subunits of the respiratory chain complexes and the mitochondrial ribosome, cytochrome-c, and intron maturases [13, 14].

Owing to their endosymbiotic origin, the mitochondrial genome organization and the mechanisms of mitochondrial gene expression largely resemble the proteobacterial origin [7]. Most of the genes are arranged in operons and transcribed as polycistronic mRNAs. Transcription is performed by two nuclear encoded T3/T7 bacteriophage type RNA polymerases, *RpoTm* (targeted to mitochondria) and *RpoTmp* (dually targeted to both mitochondria and plastids). These polymerases execute the steps of promoter recognition, transcription initiation and elongation in mitochondria [15–17]. Once the primary transcript is produced, it undergoes several maturation and processing steps, like *cis*- and *trans*-splicing, RNA trimming and editing [18]. Translation in mitochondria is carried out by membrane-bound ribosomes. These ribosomes are associated to the inner-mitochondrial membrane through multiple tethering factors to facilitate co-translational insertion of hydrophobic proteins into the membrane, thereby minimizing their exposure to the hydrophilic environment of the mitochondrial matrix [19, 20]. During translation, multiple ribosomes are associated with an individual mRNA molecule, often referred to as polysomal or polyribosomal complex. The quantification of polysomal mRNAs is commonly used as a proxy for translational activity [21, 22]. However, the firm association of mitochondrial ribosomes to the inner membrane [19, 20, 23, 24] complicates mitochondrial polysomal mRNA quantification.

Many studies were conducted to elucidate the mechanisms that govern expression of the plant mitochondrial genome. In wheat and maize, higher levels of mitochondrial transcripts were reported from the leaf base as compared to the leaf tip [25, 26] and higher levels of *atp6* and *rrn26* transcripts were reported in root meristem as compared to the root cap [27]. Furthermore, variations in mitochondrial transcript levels were also revealed in seeds during imbibition [28], suggesting a transcriptional control of mitochondrial gene expression in higher plants. Other studies reported 2- to 14-fold higher expression of mitochondrial ribosomal RNA as compared to protein-coding genes [29, 30]. These differences were assumed to result from enhanced stability of structural ribosomal RNAs [31] as compared to protein-coding mRNAs.

In *Arabidopsis*, variations in mitochondrial transcriptional activity were revealed by comparing samples collected throughout a diurnal cycle [32]. Furthermore, comprehensive run-on transcriptional analysis of mitochondrial encoded genes showed distinct transcription rates for genes encoding components of the same multi-subunit complex. These differences were not reflected in the corresponding RNA pools, suggesting significant post-transcriptional control of mitochondrial gene expression [31]. Further evidence for differential expression of mitochondrial genes was deduced from experiments using selective chemical inhibition of either the alternative or the cytochrome c pathway of the mitochondrial electron transport chain. These treatments induced opposite effects on the expression of various mitochondrial genes indicating that the mitochondrial redox state influences gene expression [33]. Also transcriptional activity in the nucleus was shown to effect mitochondrial gene expression. Parallel profiling of mitochondrial and nuclear transcripts in *Arabidopsis* seeds revealed a coordinated expression of mitochondrial and nuclear genomes to maintain the stoichiometric composition of mitochondrial electron transport chain complexes [34–36]. All investigations advocate that mitochondrial transcription and translation are dynamic processes and that both transcriptional and post-transcriptional processes are required to control the abundance of mitochondrial mRNAs in higher plants.

Not only RNA abundance, but also protein abundance varies in response to growth and environmental variations in mitochondria as it was documented for maize [37, 38], rice [39, 40], sugar beet [41], *Nicotiana sylvestris* [42], petunia [43] and *Arabidopsis thaliana* [34]. In these studies, increased mitochondrial protein synthesis was observed in leaves, flowers, shoots [41, 43], and seeds [34], while a decrease was reported in roots [41], after heat [39, 44], cold [40], chloramphenicol, erythromycin, cycloheximide [39] and methomyl treatments [45]. Due to differences in the stability or life time of different proteins, the reported variations in protein abundance cannot directly be used as a measure of translational activity. In yeast, gene-specific variation in the translational activity of mitochondrial encoded proteins was described to require specific translational activator proteins [46, 47]. In plants, only indirect evidence exists for such regulation, which is generally ascribed to a role of the 5′-untranslated leader sequence in the initiation of translation [48, 49], but experimental evidence is lacking. Nevertheless, mitochondrial ribosomes were shown to differentially translate mitochondrial transcripts [35], but the underlying regulation mechanisms are unknown.

To characterize the contributions of transcriptional and translational control to gene expression in higher plant mitochondria, we established an oligonucleotide-based microarray platform for *Nicotiana tabacum* choosing oligonucleotide sequences that could also be used to hybridize with *Arabidopsis thaliana* mitochondrial transcripts. Subsequently, a mitochondrial polysome isolation protocol was established for *N. tabacum* to analyse the polysomal mRNA abundance as a proxy for translational activity. Microarray profiling of free and polysomal mRNAs was used to investigate changes in the levels of transcripts and ribosome-bound mRNAs in tobacco leaves, roots, flowers and floral buds. In exemplary cases, western blot analysis was performed to correlate free and polysomal mRNAs with the abundance of the corresponding protein. Our data show that binding of mitochondrial mRNAs to ribosomes correlates with the total transcript level of a gene, while the abundance of tested COX2 protein does not.

## Methods

### Plant material

*Nicotiana tabacum*, cv. Petit Havana, was grown on a fertilized peat-sand mixture in the greenhouse, with 16/8 h light/dark cycle at 25/20 °C, respectively. During the day, the light intensity was maintained at 200 μE m^−2^ s^−1^. From these fertilized peat-sand grown plants, various organs (i.e. young leaves (first true leaf next to leaf primordium); fully expanded leaves (fully expanded non-senescent leaf); flowers (fully opened); and unopened floral buds were sampled as illustrated in Additional file 1: Fig. S1 Root samples were collected from plants grown hydroponically, using the following media compositions; 1 mM NH_4_NO_3_, 1 mM KH_2_PO_4_, 1 mM MgSO_4_, 250 μM CaCl_2_, 0.1 mM Fe-EDTA, 50 μM KCl, 0.1 mM H_3_BO_3_, 10 μM MnSO_4_, 2 μM ZnSO_4_, 1.5 μM CuSO_4_, and 0.1 μM Na_2_MoO_4_. These cultures were maintained in 10 l tanks, which were placed in a climate chamber with light/dark cycle of 12/12 h; light intensity 150 μmol s^−1^ m^−2^; temperature 20/18 °C and 75% relative humidity. The hydroponic nutrient solution was replaced every week during the first 3 weeks of culturing and twice a week in the following weeks.

### Design and production of mitochondrial microarrays

An oligonucleotide microarray representing all mitochondrial genes and conserved open reading frames of both *Nicotiana tabacum* (GenBank accession number BA000042.1) and *Arabidopsis thaliana* (GenBank accession number Y08501.2) was designed, using the published sequence annotations [6, 50]. The length of the oligonucleotides was chosen between 68 and 71 nucleotides and whenever possible, the GC content was adjusted close to 50%. The number of mismatches between the *N. tabacum* and *A. thaliana* oligonucleotides was not higher than a maximum of three and their position was chosen as close to either the 3′ or 5′ end as possible because mismatches close to the center of an oligonucleotide are known to have a strong destabilizing effect on the duplex [51, 52]. The final oligonucleotide sequence was adjusted for RNA editing at those sites that had been described [6, 50]. The sequence of forty-four genes showed such a high similarity between the two species that a single oligonucleotide would hybridize with cDNAs obtained from both species. In these cases, only one oligonucleotide was spotted, derived from the *Arabidopsis* sequence, to represent both species. For 25 genes [*ccb203, ccb206, ccb256, ccb382, ccb452, rps7, rps13, rps14, rps19, sdh3, atp6*-*1, apt6*-*2, orf101b, orf106b, orf11c, orf116, orf121a, orf160, orf130, trnE(uuc), trnF(gaa), trnH(gug), trnI(cau), trnP(ugg)* and *trnY(gua)*], it was not possible to design a common oligonucleotide that met all the criteria, and thus separate oligonucleotides were designed here. In total, 81 oligonucleotides were selected representing *Nicotiana tabacum* and *Arabidopsis thaliana* mitochondrial genomes (Additional file 6: Table S1). To ensure reliable quantification of the signals and comparability between arrays, a series of reference and control DNA spots were also printed on the array (Lucidea Universal ScoreCard, GE Healthcare, Buckinghamshire, UK). These reference spots are comprised of artificial genes selected from yeast intergenic regions that do not cross-hybridize with RNA from most species tested (human, mouse, rat, yeast, plant and bacteria). The Piezorray robot device (PerkinElmer, Waltham, USA) was used for printing 100 pg of each oligonucleotide onto SuperEpoxy 2 DNA Substrates (ArrayIt, Sunnyvale, USA). To avoid variations in signal intensities resulting from the differences in the local background signals, the spotting scheme was randomized and printed six times on one glass slide.

### Isolation of mitochondrial ribosome-bound mRNA

**Fig. 1:**
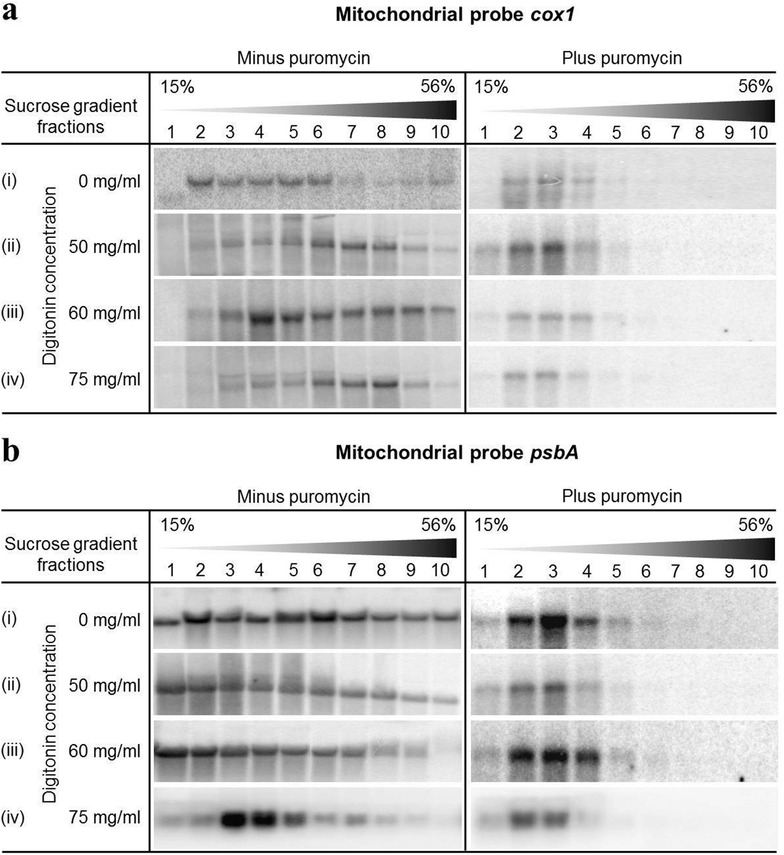
Optimization of the digitonin concentration for mitochondrial polysome isolation. To improve the extraction efficiency of the membrane-bound mitochondrial ribosomes, the detergent digitonin was added to the extraction buffer. The extraction efficiency of mitochondrial ribosomes was checked by hybridization to a specific probe for the mitochondrial cox1 gene (**a**). As the original protocol had been optimized for the isolation of plastidial polysomes, the influence of increased digitonin concentrations on plastidial ribosomes was determined by using a psbA gene specific probe as a control (**b**). Blots under the heading “mitochondrial probe cox1” (**a**) and “plastid probe psbA” (**b**) indicate two sets of parallel experiments for the optimization of digitonin concentrations. Blots under the  label “minus puromycin” indicate the polysome purification experiment and those under the label “plus puromycin” are controls to identify light and heavy polysomal fractions. In both **a** and **b** set of blots (i) represents polysome isolation procedure using a protocol suitable for plastidial polysomes where lanes 1–10 represent the different polysomal fractions. (ii) Polysome isolation by supplementing the extraction buffer with 50 mg/ml digitonin. (iii) Polysome isolation by the addition of 60 mg/ml digitonin to the extraction buffer. (iv) Polysome isolation using 75 mg/ml digitonin in the extraction buffer

To analyze mRNA bound to ribosomes, polysomes were isolated as described earlier [53] with some modifications as described below. Tissue was ground in liquid nitrogen and suspended in polysome extraction buffer (sucrose 200 mM, triton X-100 1% [v/v], polyoxyethylen-10-tridecylether 2% [v/v], heparin 0.5 mg/ml, β-mercaptoethanol 100 mM, chloramphenicol 100 μg/ml, cycloheximide 25 μg/ml). In addition, to release polysomes from the mitochondrial membrane, 60 mg/ml digitonin was used (if not stated otherwise in the text) as detergent in the extraction buffer. Cell debris was removed by two times centrifugation at 13,200*g*. The supernatant was then loaded on top of an analytical sucrose gradient (56, 40, 30 and 15% [w/v] sucrose in buffer TKM [0.4 M Tris, 0.2 M KCl and 0.1 M MgCl_2_ (HCl) pH 8.5]) and centrifuged at 272,840*g* at 4 °C for 80 min. Each gradient was separated into ten fractions of equal volumes using a fraction collector (BioRad, Hercules, USA). RNA from all fractions was extracted with phenol/chloroform. As a control treatment to distinguish fractions containing free RNA (c.f. Fig. 1, lane 1–5) from those containing ribosome-bound RNA (c.f. Fig. 1, lane 6–10), ribosomes were dissociated by adding 1.2 mM of puromycin [54] to the polysome extract prior to loading it on the sucrose gradient. For microarray analysis, RNA from the polysomal fractions (fractions 6–10) was pooled and purified with two additional precipitation steps: firstly, RNA was precipitated with 0.5 vol. 8 M LiCl at 4 °C overnight to remove residual heparin from the solution, which could inhibit the reverse transcriptase in a subsequent cDNA synthesis step [55]. Secondly, lithium ions were removed by an additional ethanol precipitation.

### Isolation of nucleic acids and RNA gel blot analysis

Total plant DNA was extracted from tobacco tissues using the cetyltrimethylammoniumbromide (CTAB)-based method [56]. Total cellular RNA from tobacco tissues was extracted using the TriFast reagent (Peqlab, Erlangen, Germany) following the manufacturer’s protocol. For Northern blot analysis, RNA samples (5–10 µg) were resolved in a 13% (v/v) formaldehyde 1% (w/v) agarose gel and transferred onto Hybond nylon membranes (GE Healthcare, Buckinghamshire, UK) by capillary blotting [57]. The membrane was hybridized to [^32^P]dCTP radiolabeled probes, generated by labeling PCR fragments specific for *cox1* (amplification primers: 5′-AGATACCCGTGCCTACTTCAC-3′ and 5′-CGACCACGAAGAAACAACAAATCC-3′), *psbA* (5′-ATAGACTAGGCCAGGATCTTAT-3′, 5′-ATTTTACCATGACTGCAATTTTAGAG-3′), *atp9* (5′-TGTTAGAAGGTGCAAAATCAATGG-3′, 5′-AACGGACTTGGAATACGAATGAGA-3′), *rps10* (5′-GACCACCAAGATAGGCATAG-3′, 5′-AAGGGTCAACGCAAGGAT-3′), *16* *s rRNA* (5′-CAAGCGGTGGAGCATGTGG-3′, 5′-GGCGGTGTGTACAAGGCCC-3′) and *18s rRNA* (5′-ACCCCAGTCGAAGACCCCACC-3′, 5′-CGCCCGAAGCATCGGACCAA-3′) with the Megaprime DNA labeling system (GE Healthcare, Buckinghamshire, UK). Hybridizations were performed at 65 °C in Church buffer (1% BSA, 1 mM EDTA, 7% SDS, 0.5 M Na_2_HPO_4_, pH 7.2) [58]. To detect the hybridized radioactive probes, membranes were exposed to storage Phosphor Screens (GE Healthcare, Buckinghamshire, UK). A Typhoon Trio imager (GE Healthcare, Buckinghamshire, UK) was used for autoradiography.

### Real-time quantitative PCR

Total RNA was treated with DNase using TURBO DNA-free kit (Ambion, California, USA). Five micrograms of RNA were reverse transcribed using the Superscript III reverse transcriptase kit (Invitrogen, Carlsbad, USA). Real time PCR amplification was performed in an optical 384-well plate using an ABI PRISM 7900 HT sequence detection system (Applied Biosystems, California, USA). The PCR reaction was based on sybr-green PCR core reagent kits (Applied Biosystems, California, USA). Data were analyzed using the SDS 2.3 software (Applied Biosystems, California, USA). Primer efficiency was calculated using the LinReg PCR program [59]. As the expression of the nuclear genes like *EF1α* (5′-ATGACCCAGCTAAGGGTGCT-3′, 5′-GACAGCAATGTGGGAGGTGT-3′) and *GAPDH* (5′-TGTGGACCTTACCGTAAGACTAGAGA-3′, 5′-CCCTCCGATTCCTCCTTGA-3′) was observed to be similar per unit fresh weight for all of the tissues and conditions tested, these genes were used as internal control to normalize the results. The C_T_ values for these control genes were used to normalize the C_T_ value of the *RpoT* (RNA polymerase) genes. The primer pairs used for *RpoT* genes were the same as used by Ramakers et al. [60]. When the mitochondrial DNA content per cell relative to nuclear DNA was determined, C_T_ for a mitochondrial intergenic region (between *orf112* and *cob*; primer pair: 5′-CCCGATACAAGCGAGCTAAG-3′; 5′-CGAGTTCATGTGCTTGCAGT- 3′), was normalized to the C_T_ of a nuclear non-coding region (*RpoTm*, encoding a mitochondrial RNA polymerase; [61, 62].

### cDNA probe synthesis for microarray analyses

For microarray analysis, cDNA probes were prepared by reverse transcription of 10 μg of total and polysomal RNA, spiked with 5 μg of random hexanucleotide primers and 2 μl of reference RNAs (GE Healthcare, Buckinghamshire, UK), and purified using S.N.A.P. columns (Invitrogen, Carlsbad, USA). Cy3 fluorescence dye was incorporated into the cDNA probes using the SuperScript Indirect cDNA labeling kit (Invitrogen, Carlsbad, USA). The volume of labeled cDNA was reduced through YM-10 microcons filters (Millipore, Bedford, USA). Labeled cDNA was dissolved in 130 μl microarray hybridization buffer (SDS 0.1%, formamide 25%, SSC 5×, BSA 1%, NaPP 40 mM) and denatured at 95 °C for 5 min. The denatured probe was added to the microarray slide mounted on the hybridisation station (HybArray 12, PerkinElmer, Waltham, USA).

### Microarray hybridization, washing and scanning

Prior to hybridization, microarray slides were washed with 2×  SSC + 0.1% sarcosyl for 2 min, 2× SSC for 2 min, 100 °C dH_2_O for 2 min and ice cold 100% ethanol for 2 min (ArrayIt, Sunnyvale, USA) in the HybArray 12 station (PerkinElmer, Waltham, USA). The slides were preheated at 75 °C for 5 min and cooled to 45 °C. They were kept at this temperature until the labeled and denatured (5 min 95 °C) cDNA was added to the microarray. The hybridization was performed for 15 h using a stepwise decrease of the temperature, starting at 44 °C for 1 h and decreasing one degree every hour until 41 °C. This final temperature was kept for another 12 h. At the end of hybridization, the slides were washed with microarray wash buffers I (SSC 2×, N-lauroylsarcosine sodium salt 0.1%), II (SSC 0.2×, N-lauroylsarcosine sodium salt 0.1%), and III (SSC 0.2×), to remove non-specifically bound probe. The slides were dried by centrifugation for 10 min at 1500 rpm. Cy3 fluorescence was detected by scanning the microarray slides with the FLA-8000 scanner (Fujifilm, Straubenhardt, Germany) with a resolution of 10 μm. The photo-multiplier tube detector was set to the highest possible sensitivity during the scanning process.

### Mitochondrial microarray data normalization and visualization

After scanning the microarray slides, the images were analyzed using the Genespotter software (Microdiscovery, Berlin, Germany). Gene spots were selected manually to ensure that the data evaluation was based on spot signals only without any background interference. Mean pixel values obtained after local background subtractions were quantified using a calibration curve from Lucidea Universal ScoreCard controls (GE Healthcare, Buckinghamshire, UK). Reference RNA that was added to the RNA samples allowed signal quantification, as the signal intensities of the reference cDNAs were plotted against the corresponding RNA amounts to record a calibration curve (Additional file 3: Fig. S3). It was then possible to calculate the relative expression values to ensure comparability between independently hybridized arrays. Normalization of the signal intensities from various slides was performed by dividing the mean pixel values with the factor obtained by calculating the slope of the calibration spots. The data obtained were multiplied by the difference in median of the various replicates. These median corrected values were used to calculate the mean values of the biological replicas and were expressed either as RNA abundance per microgram of total RNA and/or log2 ratios. The TIGR MeV software [63] and Microsoft Excel were used for data analysis and visualization. The normalized microarray data was used for the estimation of significant changes with a 2-way ANOVA (*p* < 0.05) using SigmaPlot.

### Preparation of mitochondria

Mitochondria were isolated as described by [64]. Briefly, tissues were harvested and homogenized in BoutHomX buffer (0.4 M sucrose, 50 mM Tris, 1 mM EGTA, 10 mM KH_2_PO_4_, 0.1% PVP-630, 1% fat-free BSA, 5 mM β-mercaptoethanol, pH 7.6 with HCl). The homogenate was filtered and centrifuged at 3800*g* for 5 min. The tissue debris was discarded. Mitochondria were enriched by two additional steps of centrifugation at 22,000*g* and 18,000*g* for 15 min. The resulting pellet was resuspended in gradient medium (0.5 M sucrose, 1% BSA-fat free) and layered on top of Percoll gradients (10 ml of 18% [v/v], 15 ml of 26% [v/v], and 30 ml of 50% [v/v] Percoll in 0.5 M Sucrose and 1% BSA). After centrifugation for 12 min at 40,000*g*, mitochondria were collected from the 26/50% interphase. To remove the Percoll, purified mitochondria were washed with BoutWashY (0.4 M mannitol, 10 mM KH_2_PO_4_, 0.1% BSA, pH 7.2 with KOH) and centrifuged at 17,000*g* for 10 min. The pellet was resuspended in BoutWashY and stored at − 80 °C.

### Western blot analysis

Mitochondrial proteins were extracted according to published protocols [65] with a few modifications. The extraction buffer was supplemented with 0.02% N-lauroylsarcosine and 0.02% Triton X-100 [66]. 0.75, 1.5, 3 and 6 µg of mitochondrial proteins were separated by electrophoresis on 15% SDS-containing polyacrylamide gels. The gels were either stained with Coomassie Brilliant Blue (Serva, Heidelberg, Germany) or blotted onto polyvinylidene difluoride membranes (Hybond-P, GE Healthcare, Buckinghamshire, UK). For blotting experiments, the separated proteins were transferred onto polyvinylidene difluoride membranes (Hybond-P; GE Healthcare, Buckinghamshire, UK) using the tank blot system (Perfect Blue Web M, PeqLab, Erlangen, Germany) and a standard transfer buffer (25 mM Tris, 192 mM glycine, pH 8.3). Membranes were treated with blocking buffer (20 mM Tris–HCl, pH 7.6, 137 mM NaCl, 0.1% Tween 20, and 0.5% BSA) overnight and subsequently incubated for 1 h with COX2 monoclonal antibody, diluted in buffer (20 mM Tris–HCl, pH 7.6, 137 mM NaCl, and 0.1% Tween 20). COX2 antibody dilution was 1:1000. Secondary rabbit anti-mouse IgG conjugated to horseradish peroxidase were detected with the ECL Plus proteins gel blotting detection system (GE Healthcare, Buckinghamshire, UK).

## Results

### Establishment of a mitochondrial microarray and polysome isolation protocol

To analyse mitochondrial transcript levels, we established a mitochondrial genome specific microarray that contains 81 oligonucleotides covering all genes and conserved open reading frames of the *Nicotiana tabacum* and *Arabidopsis thaliana* mitochondrial genomes (Additional file 6: Table S1). First, the microarray spotting and hybridization conditions were optimized to check if a linear relationship exists between signal intensity and RNA concentrations. To achieve this, a series of negative and reference DNAs were spotted on the array that are part of the Lucidea Universal ScoreCard controls and cover a range of 1 pg to 10 ng of DNA for quantification. The data from these references revealed that a linear relationship exists between the signal intensities and the corresponding RNA amounts (Additional file 3: Fig. S3). Next we observed that most of the RNA species represented on the microarray are quantifiable except rRNAs and tRNAs. The rRNAs were difficult to quantify, since their corresponding spots were always found to be fully saturated, obviously because the rRNAs are at least 10 to 50 times more abundant than mRNAs. Many tRNA sequences were almost identical to those encoded by the plastid genome (Additional file 8: Table S2), which precluded interpretation of variations in signal intensities. Therefore, the spotting and hybridization conditions were optimized to quantify the mRNAs and the data for rRNAs and tRNAs were not included in the analyses.

To analyse mRNAs bound to ribosomes, polysomes were isolated and the mRNA bound to them was quantified using the above mentioned microarrays. To isolate poly-ribosomal mRNAs, a sucrose density gradient centrifugation method was employed [53] and the distribution of mRNAs between the light and heavy fractions of the density gradient was determined by Northern blots (Fig. 1). Firstly, the same conditions were used as described for the isolation of plastid polysomes [53], but the resulting data revealed only very weak signals for mitochondrial *cox1* mRNA in the heavier density gradient fractions (Fig. 1a–i; fractions 6–10), while the signals for plastidial *psbA* mRNA were found fairly distributed throughout the gradient (Fig. 1b–i; fractions 6–10). This suggested that the existing protocol is unsuitable for the extraction of mitochondrial polysomes and additional modifications were needed to make the protocol suitable for the isolation of mitochondrial poly-ribosomal complexes that are known to be largely membrane bound [19, 20]. To do so, the extraction buffer was supplemented with three different concentrations of the detergent digitonin (50, 60, 75 mg/ml, respectively). Upon addition of 50 mg/ml digitonin to the extraction buffer, we observed an increase in *cox1* signal in the heavy fractions of the density gradient (fractions 6–10), which was further enhanced upon adding 60 mg/ml digitonin (Fig. 1a, ii–iii). However, when 75 mg/ml of digitonin was added to the extraction buffer, the signal for the *cox1* mRNA was shifted towards the lighter gradient fractions (Fig. 1a, iv). Likewise, the gradual increase in the digitonin concentration resulted in a decrease in the plastidial *psbA* signal in the heavier gradient fractions (Fig. 1b, ii–iv), indicating that increased digitonin concentrations result in the release of ribosomes from plastidial mRNAs. Based on these observations, 60 mg/ml of digitonin was considered optimal for the release of mitochondrial poly-ribosomes from the inner mitochondrial membrane.

### Mitochondrial gene expression in tobacco organs

**Fig. 2:**
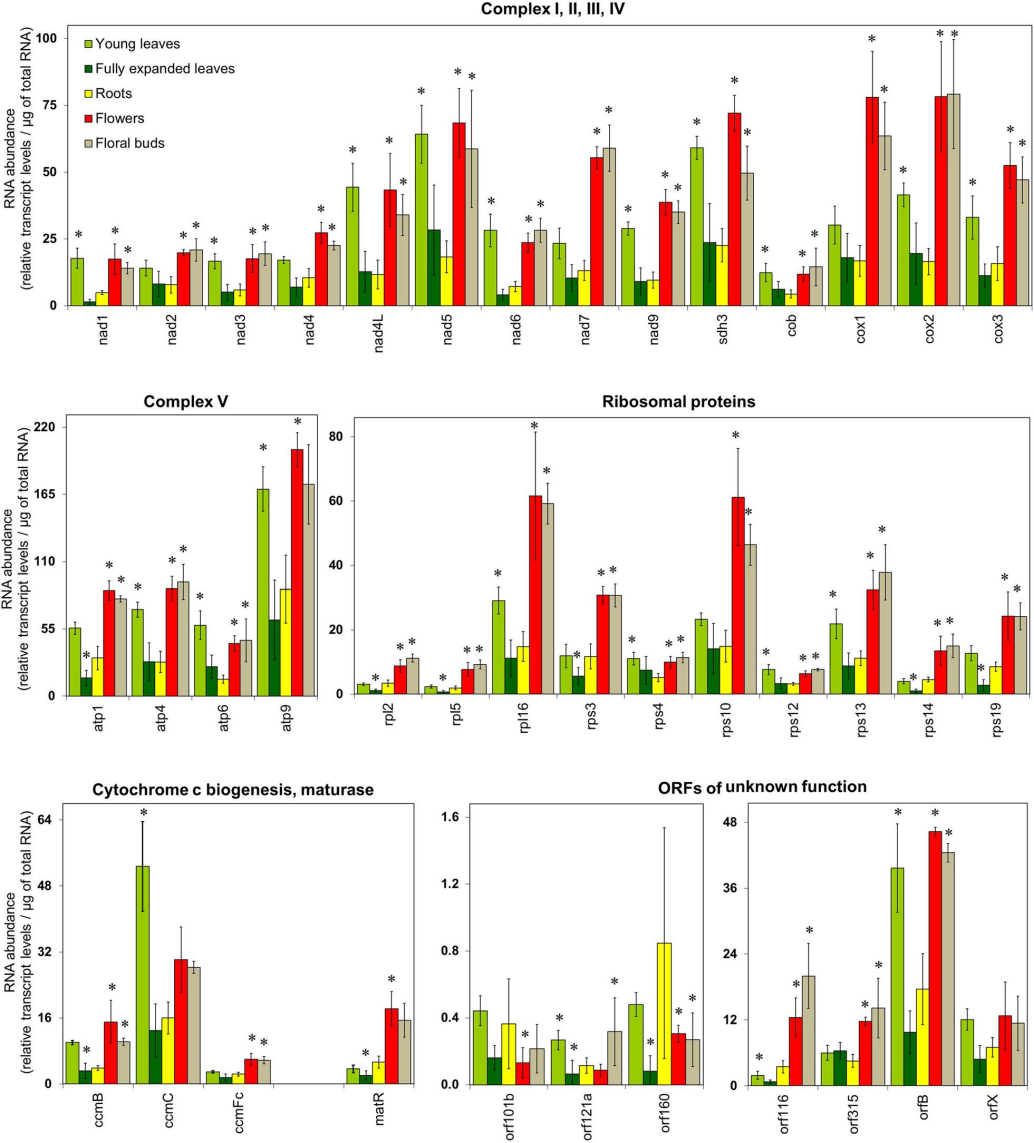
Mitochondrial RNA abundance in tobacco organs. RNA levels of mitochondrial transcripts in young leaves, fully expanded leaves, roots, flowers and floral buds. Values represent relative transcript levels per µg of total RNA (Additional file 5: Dataset 1). Data represent mean values of four biological replicates for young leaves and fully expanded leaves, three biological replicates for roots and flowers, and two biological replicates for floral buds. Significant changes were calculated using Two way ANOVA (*p* < 0.05). Significant changes relative to roots are marked by asterisks (*)

**Fig. 3:**
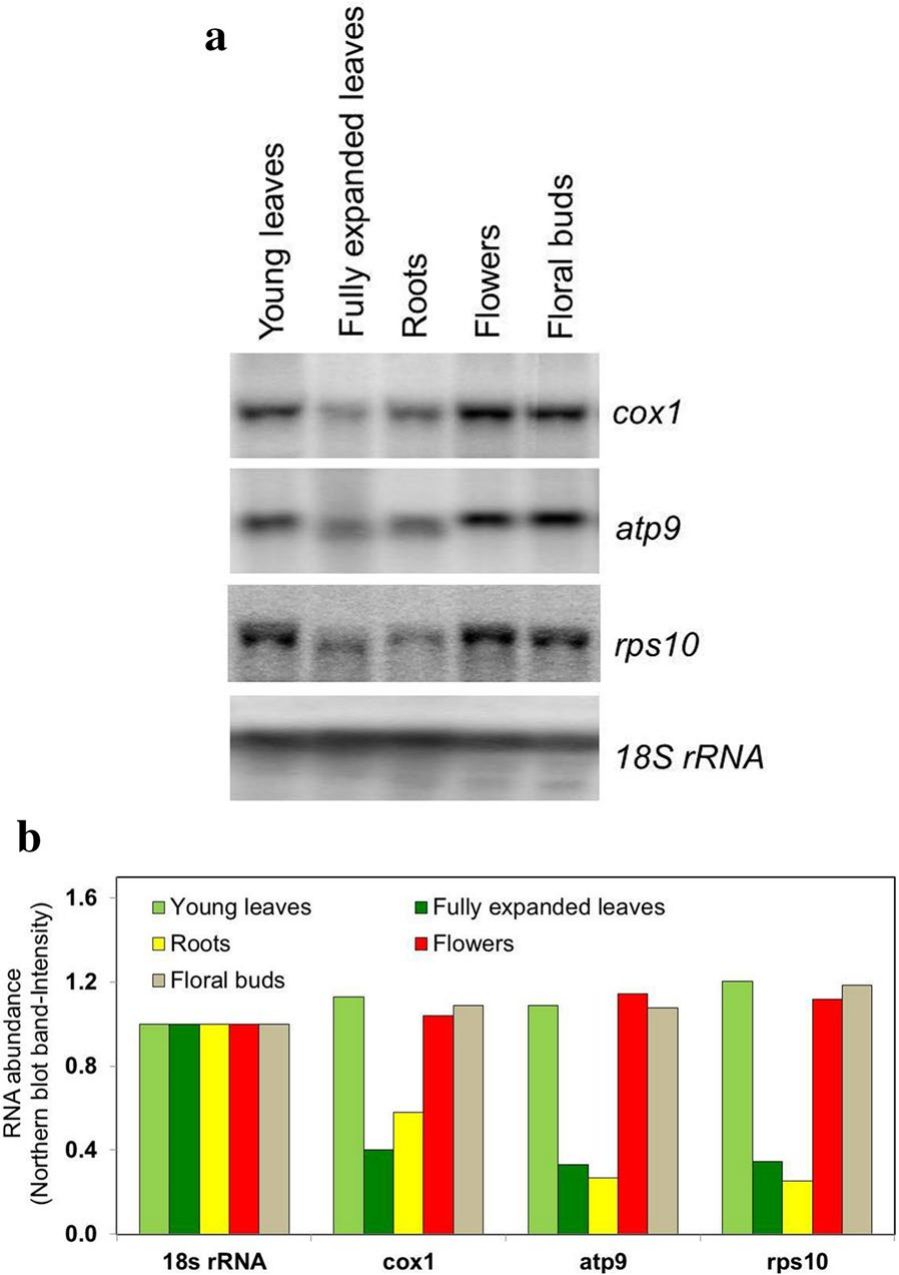
Northern blot analysis of mitochondrial RNA abundance in tobacco organs. **a** The analyzed tissues are indicated above the blots. Three mitochondrial genes (cox1, atp9 and rps10) were analyzed to confirm the pattern of mitochondrial mRNA abundance observed through mitochondrial microarray analysis. Equal loading of RNA was checked through 18S rRNA hybridization. The blots are representatives for three independent replicates. **b** Signal intensities relative to the 18s rRNA control from the Northern blots presented in part a of this figure. Pixel intensity values as determined using ImageJ were used to calculate the ratios

Changes in the mRNA abundance of mitochondrial encoded genes were investigated in young leaves, fully expanded leaves, roots, flowers and floral buds of tobacco. To this end, signal intensity values were normalised per µg of total RNA extracted from the tissue (Fig. 2; Additional file 5: Dataset S1). Moreover, to compare the differences in transcript abundances between these tissues and to mark significant changes, the data for each tissue was used as a reference to calculate ratios and presented as a heat map in a log2 scale (Additional file 2: Fig. S2). Analysis of the transcript levels revealed that mitochondrial transcripts were more abundant in flowers and floral buds as compared to fully expanded leaves and roots (Fig. 2; Additional file 2: Fig. S2b, c). In young leaves, transcripts for *nad7, cox1, rpl2, rpl5, rps3, rps10, rps14, matR, orf116, and orf315* were lower in abundance compared to flowers and floral buds, whereas *orf101b* and *orf121a* transcripts were higher in abundance than in flowers. The other transcripts have very similar levels in young leaves as observed in flower and floral buds (Fig. 2; Additional file 2: Fig. S2d–e). In roots, transcripts were lower in abundance as compared to young leaves, except for *nad2, nad4, nad7, cox1, atp1, rpl2, rpl5, rps3, rps10, rps14, rps19, ccmFc, matR, orf101b, orf116, orf160, orf315,* and *orfX* which were not significantly different in abundance between both organs (Fig. 2; Additional file 2: Fig. S2a). In fully expanded leaves, transcripts for *atp1, rpl2, rpl5, rps3, rps14 rps19, matR, orf116, orf121a* and *orf160* were lower in abundance as compared to roots, while all other transcripts had similar abundances (Fig. 2; Additional file 2: Fig. S2c). To verify these observations, selected genes (*cox1*, *atp9*, and *rps10*) were additionally investigated by Northern blot analysis (Fig. 3). The pattern of transcript abundances determined by Northern blotting corresponded exactly to the differences in expression levels that had been observed in our microarray analyses (cf. Figs. 2, 3).

### Mitochondrial DNA copy number per cell in different tobacco organs

**Fig. 4:**
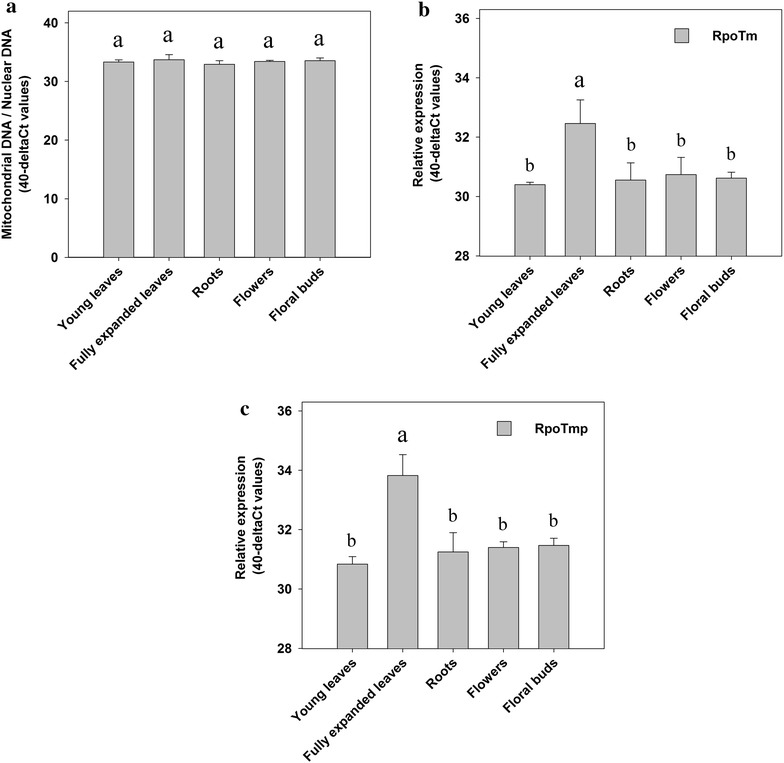
Determination of mitochondrial DNA content per nuclear DNA and the expression analysis of RpoT genes in tobacco organs using qRT-PCR analysis. Young tobacco leaves, fully expanded leaves, roots, flowers and floral buds were assayed for mitochondrial DNA content relative to nuclear DNA content (**A**). Primer combination was chosen from mitochondrial intergenic region orf112b to cob (position 34,443–40,865) and from a nuclear non-coding region from RpoTm. Data were determined by calculating the difference of mitochondrial to nuclear CT values. Bars in the figure represent standard deviations for three biological replicates. **B** RpoTm, and **C** RpoTmp messenger RNA abundance was determined by quantitative real-time PCR. Data were normalized to cytoplasmic EF1α and GAPDH levels as an internal control. RpoT gene expression is presented as 40-ΔCT values. Given values were derived from three different biological replicates; standard deviations are indicated. Different letters mark mean values that are significantly different from each other (one way ANOVA; *p* < 0.05 followed by Tukey test)

To correlate mitochondrial transcript levels with the mitochondrial DNA copy number in different tissues, the amount of mitochondrial DNA per cell was determined through quantitative real-time PCR analysis and normalised to the nuclear DNA content of the tissue. In tobacco nuclear DNA content can be used as a reference since tobacco cells are diploid and do not undergo somatic endopolyploidization. This analysis revealed that similar amounts of mitochondrial DNA are present in all tobacco organs tested (young leaves, fully expanded leaves, roots, flowers and floral buds; Fig. 4a). Therefore, it can be concluded that the variation observed in mitochondrial transcript levels (through microarray analysis and Northern blotting; Figs. 2, 3) is not caused by different abundance of mitochondrial DNA in these tobacco organs.

### Expression of *RpoT* genes in tobacco organs

To determine if a correlation exists between the expression patterns of nuclear encoded mitochondrial RNA polymerases and mitochondrial encoded genes, the expression of *RpoTm* (mitochondria) and *RpoTmp* (mitochondria and plastids) was quantified through qRT-PCR. This analysis illustrated significantly higher abundance (4–6-fold) of *RpoTm* and *RpoTmp* transcripts in fully expanded leaves relative to young leaves, roots, flowers and floral buds (Fig. 4b, c). By contrast, comparable levels of *RpoTm* and *RpoTmp* transcripts were found in young leaves, roots, flowers and floral buds. This suggests that the differences detected in mitochondrial transcript levels through microarray analysis are not primarily caused by alterations in *RpoTm* and *RpoTmp* transcript levels in these organs.

### Mitochondrial ribosome-bound mRNA abundance in tobacco organs

**Fig. 5:**
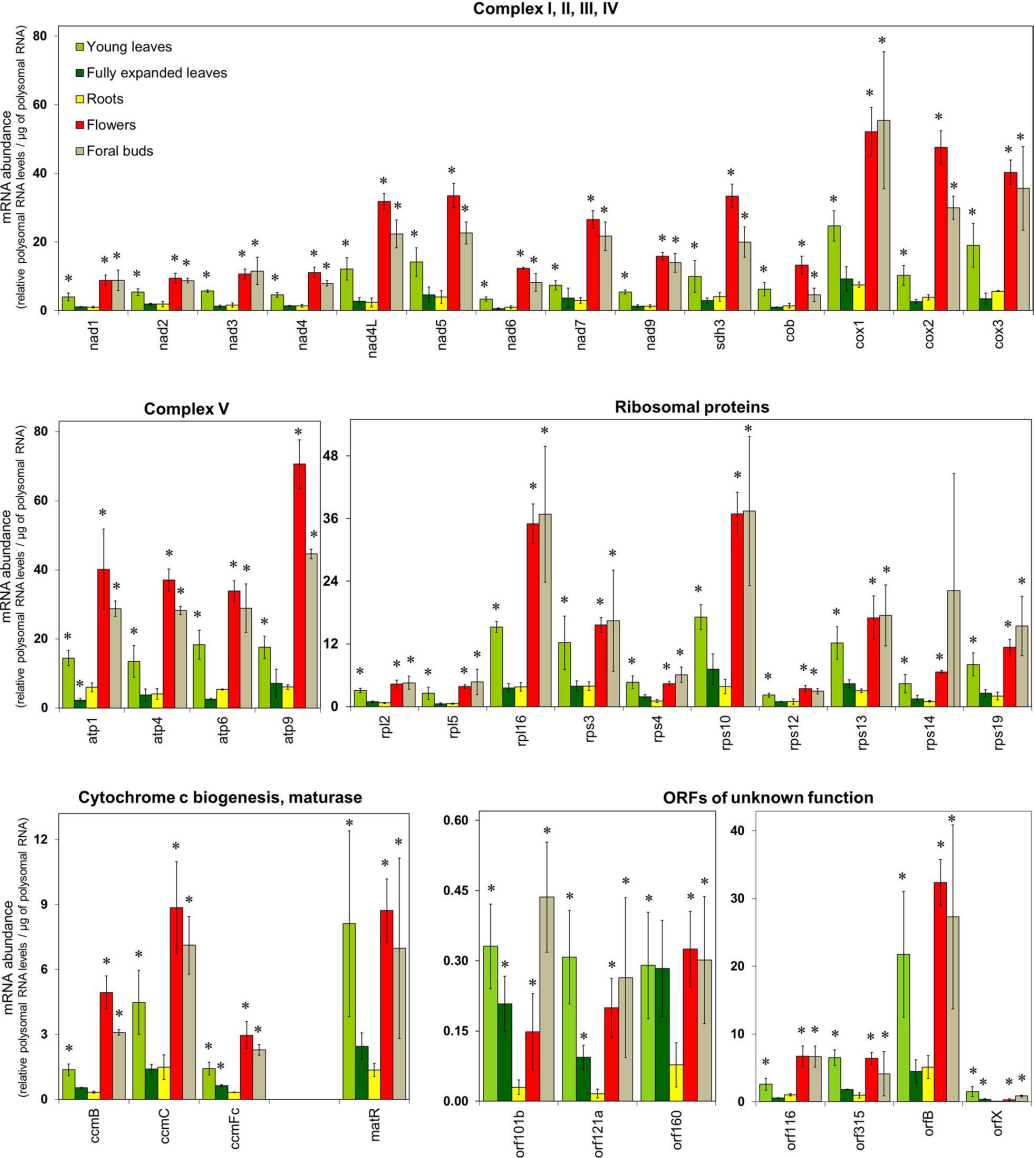
Ribosome bound mRNA abundance in tobacco organs. Abundance of mRNA bound to ribosomes in young leaves, fully expanded leaves, roots, flowers and floral buds of tobacco. Values represent relative polysomal RNA levels per µg of ribosome-bound RNA (Additional file 7: Dataset 2). Data represent mean values of four biological replicates for young leaves and fully expanded leaves, three biological replicates for roots and flowers and two biological replicates for floral buds. Significance was estimated with two way ANOVA; *P* < 0.05. Significant changes relative to the roots are marked by asterisks (*)

To analyse the abundance of mRNAs that are associated with mitochondrial ribosomes, polysomal RNA isolation was carried out for young leaves, fully expanded leaves, roots, flowers and floral buds (Fig. 5; Additional file 7: Dataset S2). Signals were obtained for all mitochondrial mRNA species in the polysomal fractions using the microarray. We observed that the levels of ribosome-bound mRNAs are higher in flowers and floral buds compared to fully expanded leaves and roots. In young leaves, the ribosome-bound mRNA levels were, on average, only half as abundant as in flowers and floral buds. Notable exceptions are *matR, orf315* and *orfB*, which were as abundant in flowers and floral buds. In fully expanded leaves and roots similar levels of ribosome-bound mRNAs were present, except for *atp1* which was lower in abundances and *ccmFc, orf101b, orf121a,* and *orfX* which were higher in abundance in fully expanded leaves compared to roots.

**Fig. 6:**
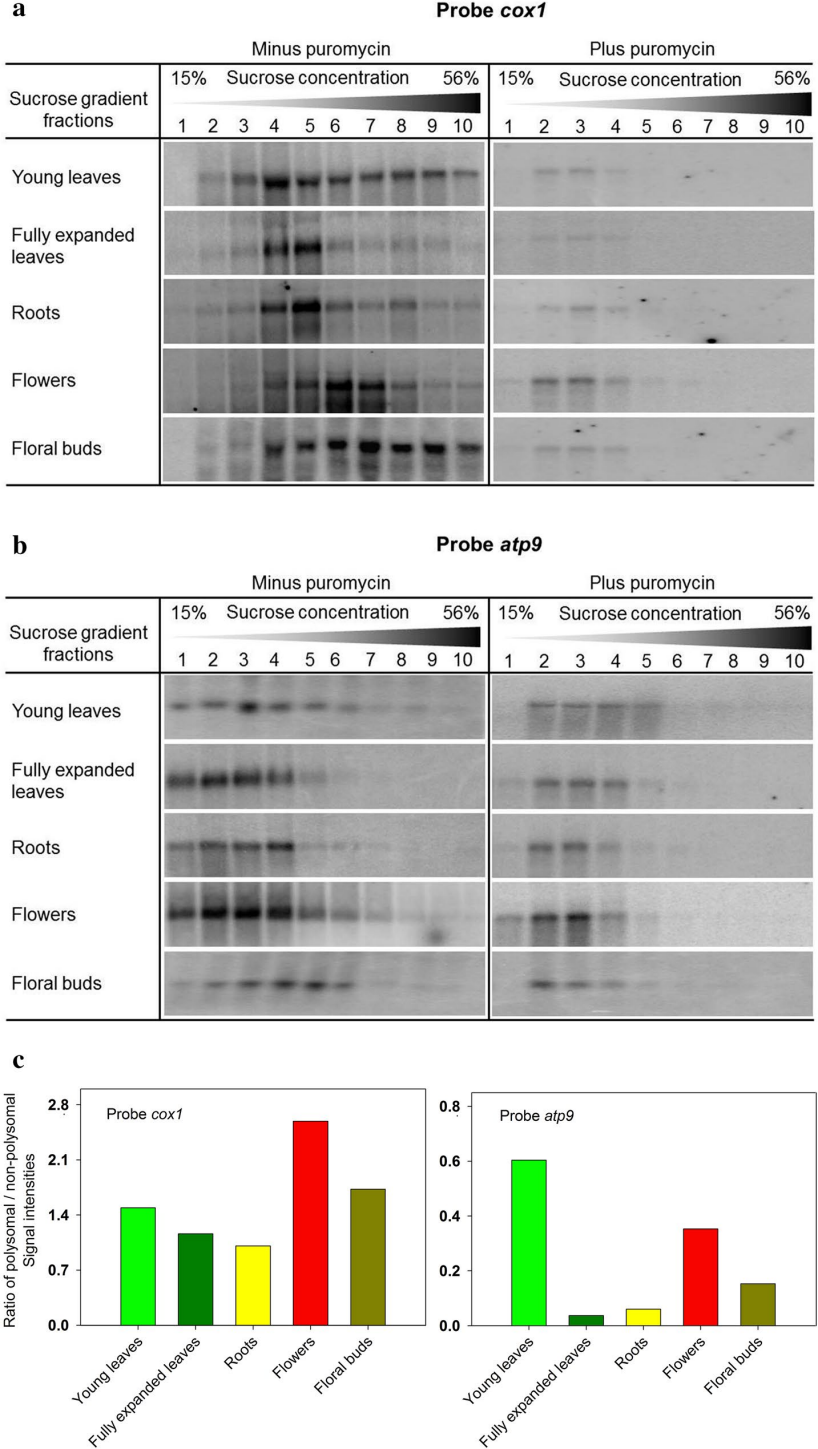
Northern blot analysis of mitochondrial ribosome-bound mRNA levels in different tobacco organs. Polysome gradients were fractionated into ten fractions, and equal aliquots of extracted RNAs were separated by denaturing agarose gel electrophoresis, blotted, and hybridized to radiolabeled probes specific for *cox1* (**a**) and *atp9* (**b**) The wedges above the blots indicate the gradient from low to high sucrose concentration. As a control, a sample was treated with puromycin to cause dissociation of ribosomes from the mRNAs. Northern blots presented here are representatives for three independent technical replicates. **c** Quantification of the band intensities of ribosome-bound mRNA shown in the Northern blots above. Values represent the ratio between polysomal and non-polysomal bands

To validate, the abundance of mitochondrial ribosome-bound mRNA levels detected through microarray analysis, Northern blot analyses of fractionated polysome gradients were carried out, using *cox1* and *atp9* gene-specific probes (Fig. 6). In these experiments, the *cox1* mRNA was found to be more abundant in the dense gradient fractions of flowers and floral buds and to a less extent of young leaves than in those of fully expanded leaves and roots (Fig. 6a). Although the *cox1* mRNA also could be detected in all polysomal fractions from fully expanded leaves and roots, the signals are much weaker than in young leaves, flowers and floral buds. Also, the abundance of *atp9* mRNA in the ribosome-bound fractions was detected to be higher in young leaves, flowers and floral buds as compared to fully expanded leaves and roots (Fig. 6b). This analysis confirms the pattern for ribosome-bound mRNA levels as observed through microarray hybridization experiments for these organs.

To visualize gene-specific differences in polysome-bound mRNA levels between the different tissues, the data for each tissue were taken as a reference to calculate ratios (Fig. 5; Additional file 3: Fig. S3; Additional file 7: Dataset S2). This analysis showed an overall significantly higher abundance of ribosome-bound mRNA levels in young leaves, flowers and floral buds compared to roots (Additional file 3: Fig. S3b). The changes in polysomal mRNA levels between fully expanded leaves and roots were negligible for most of the genes, except for *atp1* (less abundant in fully expanded leaves compared to roots), *ccmFc, orf101b, orf116, orf121a* and *orfX* (more abundant in roots compared to fully expanded leaves) (Additional file 3: Fig. S3b, c). In flowers and floral buds, polysomal mRNA levels showed a clear tendency to be higher in abundance as compared to young leaves (Additional file 3: Fig. S3a). However, the relative amount of ribosome-bound mRNA in flowers compared to floral buds and vice versa indicated for the most part little or no difference between the two organs (Additional file 3: Fig. S3d, e). Overall, this analysis confirmed that mitochondrial ribosome-bound mRNAs levels are significantly higher in young leaves, flowers and floral buds as compared to fully expanded leaves and roots.

**Fig. 7:**
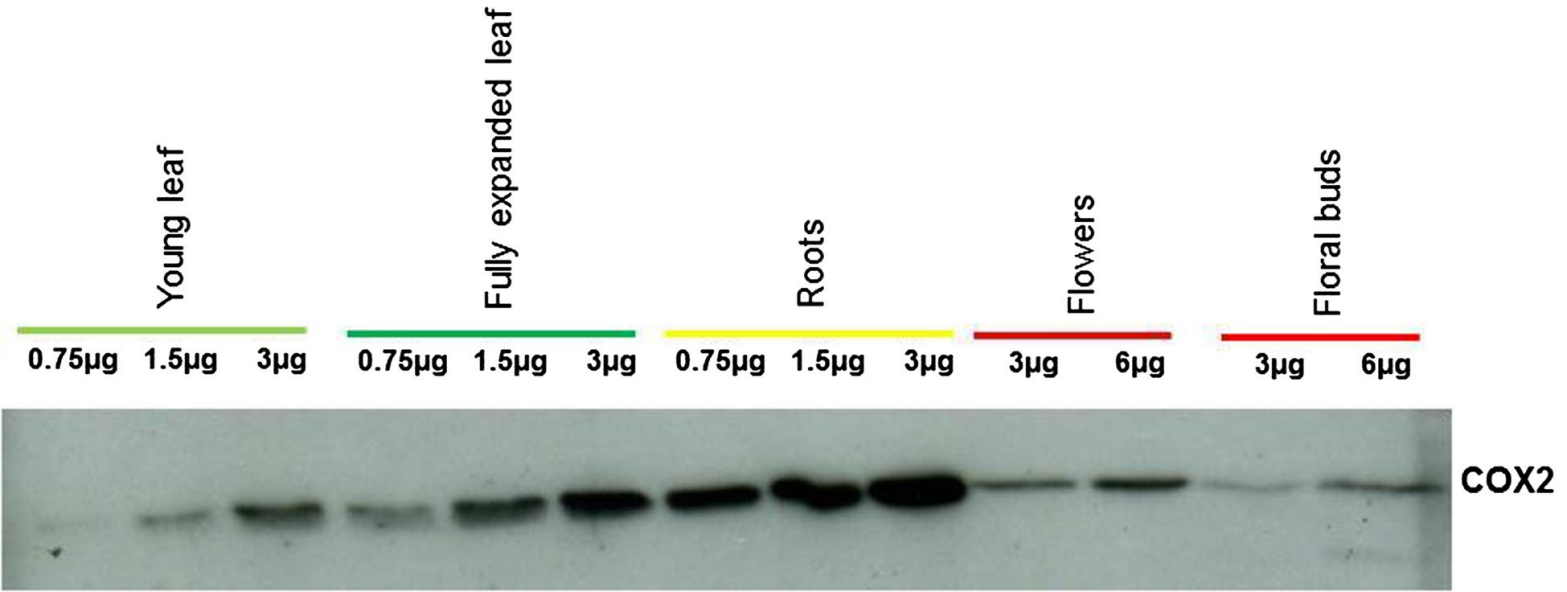
Western blot analysis to determine COX2 protein abundances in tobacco organs. Mitochondria isolated from young leaves, fully expanded leaves, roots, flowers and floral buds for western blot analysis using anti-COX2 antibody (provided by Prof. Thomas D. Fox, Cornell University, USA). For quantitative assessment of protein abundance in these tissues, dilution series of extracted mitochondrial proteins were loaded onto the gel for each tissue. Data were confirmed by analysis of three biological and technical replicates. Equal loading was further confirmed by silver staining of the gel

To determine whether the ribosome-bound mRNA levels in the different organs correspond to the actual protein levels, western blot analysis was conducted. To this end, we tested antibodies against the RPS12 [67], RPS13 [68] and COX2 [69] proteins. Only the COX2 antibody (kindly provided by Prof. Thomas D. Fox, Cornell University, USA) was found to react specifically. We observed that the COX2 protein accumulates to the highest level in tobacco roots, followed by fully expanded and young leaves. The lowest levels were detected in flowers and floral buds (Fig. 7). Remarkably, this pattern of protein abundance is exactly reciprocal to the transcript and ribosome-bound mRNA levels observed for these organs.

## Discussion

### Establishing a mitochondrial microarray and mitochondrial polysome analysis

In the course of this study, an oligonucleotide microarray platform for the *Nicotiana tabacum* and *Arabidopsis thaliana* mitochondrial genomes was developed to study changes in mitochondrial transcript levels. In total, this microarray contains 81 oligonucleotides covering all genes and conserved open reading frames (Additional file 6: Table S1). The length of oligonucleotides was fixed to 70-bases because arrays based on 70-bases long oligonucleotides were reported to be 4-fold more sensitive as compared to 50- and 60-bases long oligonucleotide arrays. Furthermore, 70-mer oligonucleotide arrays were also found to produce hybridization signals comparable to PCR amplicon based arrays [70–74]. Therefore, to detect mitochondrial transcripts at the highest possible sensitivity, we have used only one 70-mer oligonucleotide probe for all genes represented on our microarray.

To analyze mRNAs associated with ribosomes, mitochondrial polysome isolation was optimized. In yeast, the isolation of mitochondrial ribosomes was shown to be possible by supplementing the extraction buffer with ionic (2–4%, sodium deoxycholate) and/or non-ionic (0.2–0.5% triton) detergents [75–77]. Therefore, to improve the extraction efficiency of membrane-bound ribosomes from plant mitochondria, we have supplemented the extraction buffer with different concentrations (50, 60 and 75 mg/ml) of digitonin. Digitonin can readily disrupt the outer mitochondrial membrane as it contains substantial amount of cholesterol [78, 79] however, to lyse the inner mitochondrial membrane, that is devoid of cholesterol [80] high concentrations of digitonin was required for the release of mitochondrial membrane-bound ribosomes in the present study.

To observe the release of mitochondrial polysomes and to distinguish between polysomal and non-polysomal mRNA fractions of the sucrose density gradients, a series of Northern blot experiments were conducted (Fig. 1). First, the polysomal fractions were distinguished from the non-polysomal ones by treating part of the sample with puromycin prior to centrifugation (Fig. 1; plus puromycin). Secondly, to identify the optimal digitonin concentration for efficient release of mitochondrial polysomes from the inner mitochondrial membrane, different digitonin concentrations were tested. We observed that, with the initial increase in digitonin concentration from 50 to 60 mg/ml of extraction buffer, the signals from mitochondrial ribosome-bound mRNAs were enhanced in the heavy gradient fractions, but shifted to lighter gradient fractions upon further increasing the digitonin concentration to 75 mg/ml. The improved polysome yield at 60 mg/ml compared to 50 mg/ml probably can be attributed to more efficient solubilization of mitochondrial membranes, thus releasing more poly-ribosomal complexes. A further increase of the digitonin concentration to 75 mg/ml, apparently, does not only solubilize the mitochondrial membranes, but also results in partial dissociation of poly-ribosomal complexes from the mRNA, thus shifting the *cox1* signals to lighter gradient fractions (Fig. 1a). Consistent with this interpretation, the increase in digitonin concentration also resulted in the release of plastidial poly-ribosomes from the mRNA molecules, thus shifting the *psbA* signals to lighter gradient fractions (Fig. 1b). This indicates that digitonin concentrations higher than 60 mg/ml are not suitable for the isolation of polysomes. Based on the Northern blot experiments, 60 mg/ml of digitonin was chosen to release the maximum number of mitochondrial poly-ribosomal complexes from the inner mitochondrial membrane while minimizing polysome dissociation.

In the current study, we have opted to design a microarray platform rather than using Northern blotting, RT-qPCR or RNAseq, but any of these alternative techniques could have been used to evaluate polysome loading. The microarray and polysome protocol as established here allows the simultaneous monitoring of relative RNA levels and their translational state from multiple samples. In fact, the developed microarray greatly extends the biological capability of mitochondrial gene expression screening experiments. However, recently, next generation sequencing methodology has tremendously improved the detection sensitivity and accuracy of mitochondrial RNA species [81]. RNA seq analysis of mRNA associated with ribosomes has not only made it possible to precisely detect the translational expression profiles of characterized and uncharacterized protein-coding open reading frames (ORFs) but also of intergenic regions [81].

### Mitochondrial transcript levels vary strongly between tobacco organs

Comparison of mitochondrial transcript levels from young leaves, flowers, floral buds, fully expanded leaves and roots revealed lowest transcript levels in fully expanded leaves and roots and relatively high levels in floral tissue. Comparing young leaves with fully expanded leaves shows that transcript levels decrease throughout development (Fig. 2). The differences in transcript abundances among the organs could neither be explained on the basis of mitochondrial DNA content per cell in these organs and nor did it correlate with the expression level of mitochondrial RNA polymerases. Previous studies to characterize mitochondrial transcription and its regulation in wheat and maize demonstrated a similar developmental gradient within a leaf; i.e. higher expression in the basal, meristematic cells and lower expression in the senescing cells at the tip. The higher mitochondrial transcript levels as detected in young leaves, flowers and floral buds might be related to the activity of cell division, growth and differentiation in these tissues as compared to fully expanded leaves and roots [27]. This is also supported by the higher expression levels of mitochondrial genes in flowers compared to leaves in maize, and in floral tissues compared to seedlings in sunflower [82, 83].

In brief, our results demonstrate that mitochondrial transcription machinery respond to cell division, tissue growth and differentiation, not in a gene specific or functional category specific manner but rather it transcribe all genes on the mitochondrial genome. A general view of our results suggests that higher mitochondrial transcripts are present in tissues with higher cell division, perhaps reflecting the requirement for a more active mitochondrial biogenesis in these tissues. However, active mitochondrial biogenesis is not always correlated with higher mitotic activity [84], therefore, the observed differences in mitochondrial transcripts must be related to tissue specific developmental process, perhaps more related to mitotic activity of the cell rather than mitochondrial biogenesis as mitochondrial genome copies per cell remained similar in these tissues.

### The level of ribosome-bound mRNA in mitochondria is largely determined by the overall transcript level of a gene

In the present study the level of ribosome-bound mRNAs was in general higher in mitochondria from young leaves, flowers and floral buds than in fully expanded leaves and roots. In young leaves, ribosome-bound mRNA levels are lower than in flowers and floral buds (Fig. 5). Previously, organ-specific expression of mitochondrial proteins was demonstrated in several plant species using one- or two-dimensional gel electrophoresis. For example, mitochondrial proteins synthesis was shown to vary between cobs, tassels, kernel scutella and shoots in maize [38]; between green leaves, etiolated leaves and hypocotyls in pea [85]; between flowers, roots and leaves in sugar-beet [41]; and between developing pollens and leaves in *Nicotiana sylvestris* [42]. In our study, we showed for the COX2 protein that, higher ribosome-bound mRNA levels were associated with lower protein levels and lower ribosome bound mRNA levels were associated with higher protein levels. This indicates that, in plant mitochondria, the level of ribosome-bound mRNAs does not necessarily reflect the actual protein abundance, suggesting that significant regulation occurs at the post-translational level. However, since we could only obtain specific antibodies for COX2 protein, further studies are required in the future to investigate the general relation between mitochondrial protein abundance and mRNA association to the mitochondrial ribosomes.

In the present study, variation in total mitochondrial transcript levels followed similar patterns as ribosome-bound mRNAs (compare Figs. 2, 5). This implies that, in plant mitochondria, the transcript level has a strong influence on the amount of mRNA that is translated (or at least associated with ribosomes). The lifetime of individual mRNAs is known to be influenced by their association with ribosomes: in most instances, ribosomes act as protective barriers to hinder mRNA cleavage by ribonucleases [86]. Here, we have observed that organs with high mitochondrial transcript abundances also have higher ribosome-bound mRNA levels and those with lower transcript levels have lower ribosome-bound mRNA levels, possibly suggesting that mitochondrial mRNAs are stabilised by binding of ribosomes.

Although it is tempting to use ribosome binding of mRNAs as a proxy for translational activity, polysome association is not necessarily positively correlated with the actual protein abundance. This is due to the impacts of post-translational modifications, regulated proteolysis, protein folding and assembly into complexes on the lifetime of a protein. This consideration is also valid for the results presented here, where the ribosome-bound mRNA levels did not correspond to the actual protein abundance in that organs with high mitochondrial ribosome bound *cox2* mRNA levels showed low COX2 protein abundance.

Our results indicate that ribosome binding of mRNAs though signifies active translation but it does not reflect the impact of post-translational modification on the lifespan of a protein. In other words, the discrepancy observed between the ribosome bound mRNAs and the actual protein levels might have resulted from the differences in the in vivo half-life of mitochondrial encoded proteins in these organs. This means that mitochondrial proteins might have produced in the tissue revealing active ribosome loading but get degraded either due to premature chain termination, being damaged or unstable. The latter is particularly relevant to proteins that are components of the multi-protein complexes and whose stability depends on the accumulation of other subunits in stoichiometric amounts [48]. Thus, the mechanism controlling the actual protein abundance in plant mitochondria seems to be operative during and after translation.

Additionally, mitochondrial genes also contain multiple introns [37, 87, 88] and their transcripts show multiple signals by Northern analysis. Since the loading of transcripts by the polysomes is dependent on the 5ʹ flanking sequences and both spliced and un-spliced transcripts contain identical regions flanking the start codon, there seems to be no basis for the discrimination of spliced and un-spliced transcripts by the ribosomes. In fact, both spliced and un-spliced precursor mRNAs were found attached to the mitochondrial ribosomes directing the synthesis of polypeptides that prematurely terminate during the translation of intron sequences [37, 89]. For the current study, some microarray probes like the one designed for nad2 and nad5 cannot detect un-trans-spliced polysomal mRNAs, since these probes were designed to bind to downstream exons. This suggests that splicing efficiencies might affect detection of polysome associated mRNAs through established mitochondrial microarray. Therefore, the data presented here for microarray analysis of mitochondrial polysomal mRNA present signals only for fully spliced transcripts for nad2 and nad5.

Moreover, both edited and unedited transcripts are known to get attached by the mitochondrial ribosomes resulting into fully edited and partially edited proteins [67, 90, 91]. However, only fully edited proteins become part of the multi-subunit protein-complexes [90–92]. This suggests that while all mitochondrial mRNAs are translated indiscriminately of their editing status, selection occurs post-translationally due to which ribosome binding of mRNA do not reflect the actual protein abundances. In short, the observed disparities between ribosome bound mRNA and COX2 protein abundance in various tissues require future investigations to study the importance of post-translational mechanisms like protein structural modification and degradation in the regulation of translation in plant mitochondria.

## Conclusions

In conclusion, a technical platform was established to determine mitochondrial transcript levels, together with the optimization of a method to isolate mitochondrial polysomes to analyse ribosome-bound mRNAs. The analysis indicates that transcript levels vary proportionally to the ribosome-bound mRNA levels. For one protein, COX2, the actual abundance of protein was tested as well for the various tissues and appeared not to correlate with the mRNA level and the intensity of ribosome association. From our work and other studies discussed here, it is concluded that it should be considered that mitochondrial gene expression is influenced at different levels, and post-translational control mechanisms can override changes at the transcriptional and/or translational level.

## Additional files

**Additional file 1: Fig. S1.** Experimental material used for the transcriptional and translational analysis of mitochondrial gene expression in different tobacco organs. Tobacco organs [young leaves (a), fully expanded leaves (b), roots (c), flowers (d) and floral buds (e)] harvested at different stages of growth for mitochondrial transcriptional and translational analysis are shown.

**Additional file 2: Fig. S2.** Heat map of ratios of transcript abundances between tobacco organs in a log2 scale. Transcript abundances in each tissue were taken as reference which can be recognized as a blank column. (a) Ratios with young leaves as control, (b) Ratios with fully expanded leaves as control, (c) Ratios with roots as a control, (d) Ratios with flowers as a control, (e) Ratios with floral buds as a control. A log2 (ratio) > 0 is represented by blue colors and a log2 (ratio) < 0 is marked in red. Significant changes were calculated using two way ANOVA (P < 0.05) and are marked by asterisks (*) relative to the reference organ.

**Additional file 3: Fig. S3.** Heat map from ratios of ribosome-bound mRNA abundance between tobacco organs in a log2 scale. Ribosome-bound mRNA levels in each tissue were taken as a reference which can be recognized as a blank column. (a) Ratios with young leaves as control, (b) Ratios with fully expanded leaves as control, (c) Ratios with roots as a control, (d) Ratios with flowers as a control, (e) Ratios with floral buds as a control. A log2 (ratio) > 0 is represented by blue colors and a log2 (ratio) < 0 is marked in red. Significant changes were calculated using two way ANOVA (P < 0.05) and are marked by asterisks (*) relative to the reference organ.

**Additional file 4: Fig. S4.** Linear regression of spiked in reference RNAs. Calibration curve is presented here as an example. It is based on values corresponding to the calibration samples spotted on the microarray. The factor obtained from the slop of this graph was used to normalize the microarray data to remove the differences that might result from differences in hybridization efficiencies.

**Additional file 5: Dataset S1.** Mitochondrial transcript levels in tobacco organs [(total RNA (AVG) and standard deviations) and log2 fold change in expression of total RNA]. The data represent mean values of four biological replicates for young leaves and fully expanded leaves, three biological replicates for roots and flowers and two biological replicates for floral buds. The log2 fold change is the transformation of ratios taking data for each tissue as a reference.

**Additional file 6: Table S1.** Mitochondrial microarray oligonucleotides for Nicotiana tabacum and Arabidopsis thaliana.

**Additional file 7: Dataset S2.** Mitochondrial polysomal mRNA levels in tobacco organs [(polysomal RNA (AVG) and standard deviations) and log2 fold change in expression of polysomal RNA]. The data represent mean values of four biological replicates for young leaves and fully expanded leaves, three biological replicates for roots and flowers and two biological replicates for floral buds. The log2 fold change is the transformation of ratios taking data for each tissue as a reference.

**Additional file 8: Table S2.** Blast results of mitochondrial tRNA oligonucleotides. Blast searches were carried out to identify the percentage of sequence similarity between mitochondrial and plastid encoded tRNA genes. The nucleotide sequence for most mitochondrial tRNAs [except for *trnE(uuc)* and *trnI(cau)*] are highly similar (70% to 100%) to those encoded by the plastids.
