# Supplementary figures and images for: Comparison of mitochondrial gene expression and polysome loading in different tobacco tissues
Source: Plant Methods. 2017 Dec 13;13:112. doi: 10.1186/s13007-017-0257-4 (PMC5729415; doi:10.1186/s13007-017-0257-4)

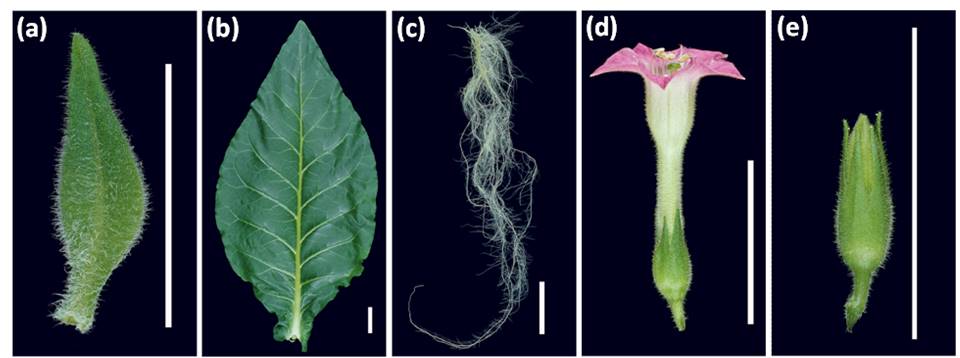

Supplement: Supplementary file 1 — Additional file 1: Fig. S1. Experimental material used for the transcriptional and translational analysis of mitochondrial gene expression in different tobacco organs. Tobacco organs [young leaves (a), fully expanded leaves (b), roots (c), flowers (d) and floral buds (e)] harvested at different stages of growth for mitochondrial transcriptional and translational analysis are shown. [file 13007_2017_257_MOESM1_ESM.jpg]

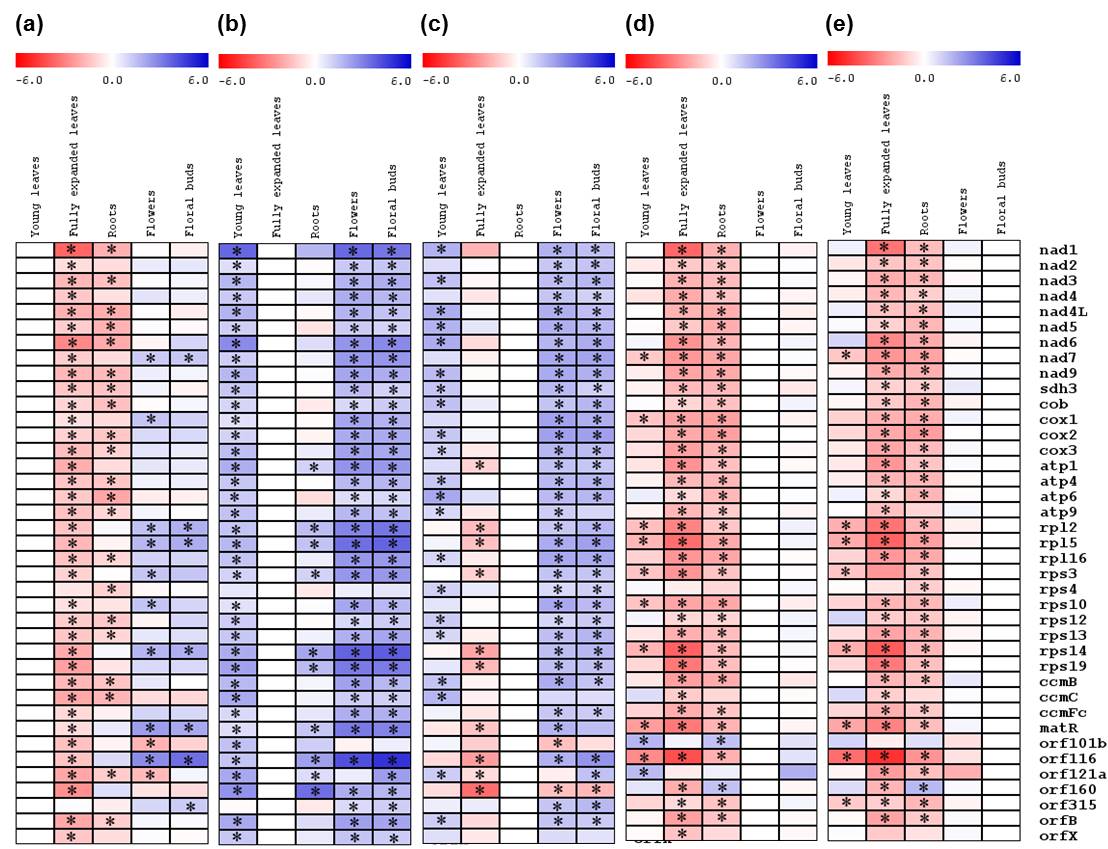

Supplement: Supplementary file 2 — Additional file 2: Fig. S2. Heat map of ratios of transcript abundances between tobacco organs in a log2 scale. Transcript abundances in each tissue were taken as reference which can be recognized as a blank column. (a) Ratios with young leaves as control, (b) Ratios with fully expanded leaves as control, (c) Ratios with roots as a control, (d) Ratios with flowers as a control, (e) Ratios with floral buds as a control. A log2 (ratio) > 0 is represented by blue colors and a log2 (ratio) < 0 is marked in red. Significant changes were calculated using two way ANOVA (P < 0.05) and are marked by asterisks (*) relative to the reference organ. [file 13007_2017_257_MOESM2_ESM.jpg]

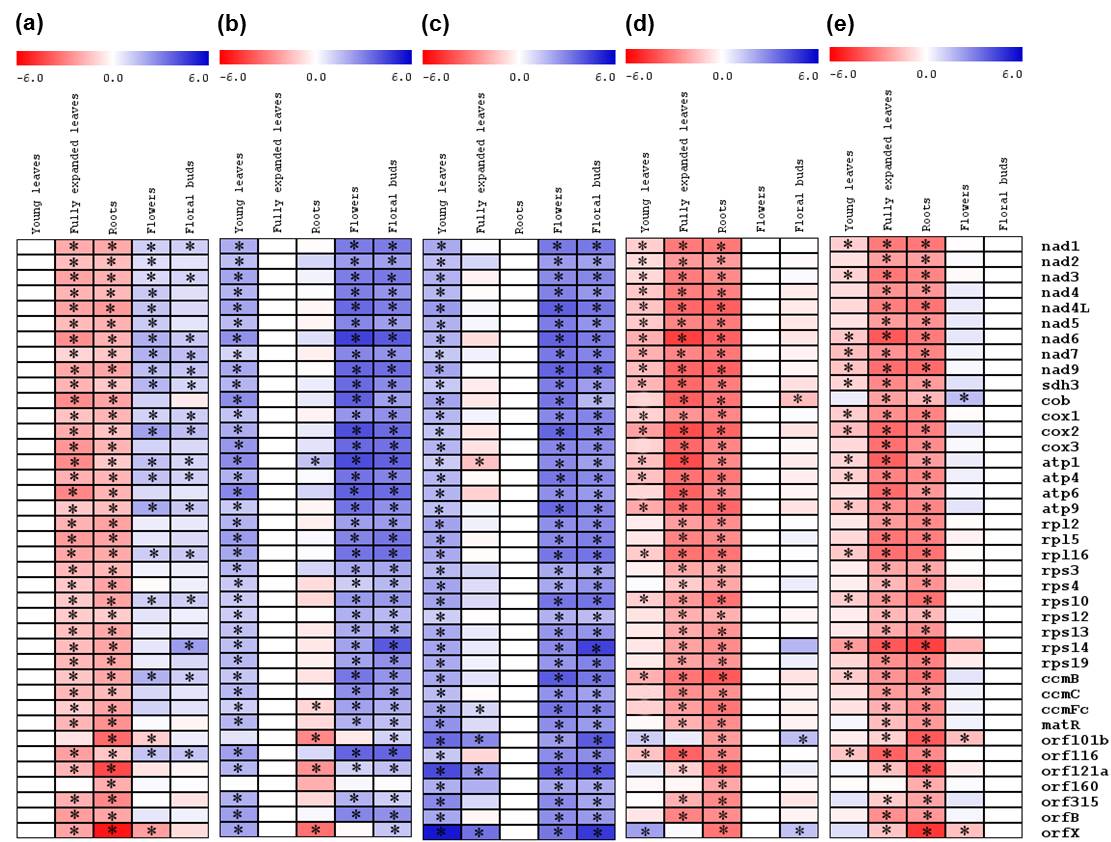

Supplement: Supplementary file 3 — Additional file 3: Fig. S3. Heat map from ratios of ribosome-bound mRNA abundance between tobacco organs in a log2 scale. Ribosome-bound mRNA levels in each tissue were taken as a reference which can be recognized as a blank column. (a) Ratios with young leaves as control, (b) Ratios with fully expanded leaves as control, (c) Ratios with roots as a control, (d) Ratios with flowers as a control, (e) Ratios with floral buds as a control. A log2 (ratio) > 0 is represented by blue colors and a log2 (ratio) < 0 is marked in red. Significant changes were calculated using two way ANOVA (P < 0.05) and are marked by asterisks (*) relative to the reference organ. [file 13007_2017_257_MOESM3_ESM.jpg]

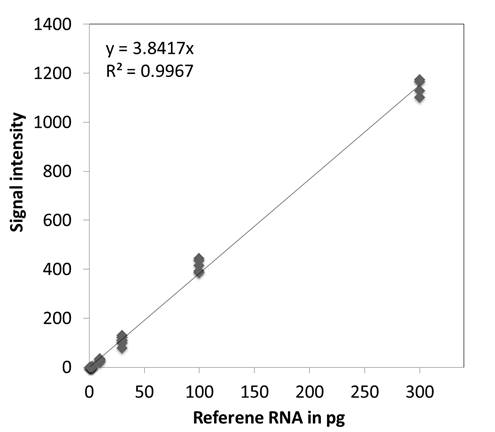

Supplement: Supplementary file 4 — Additional file 4: Fig. S4. Linear regression of spiked in reference RNAs. Calibration curve is presented here as an example. It is based on values corresponding to the calibration samples spotted on the microarray. The factor obtained from the slop of this graph was used to normalize the microarray data to remove the differences that might result from differences in hybridization efficiencies. [file 13007_2017_257_MOESM4_ESM.jpg]
